# Exergames in Childhood Obesity Treatment: A Systematic Review

**DOI:** 10.3390/ijerph18094938

**Published:** 2021-05-06

**Authors:** Federica Valeriani, Carmela Protano, Daniela Marotta, Giorgio Liguori, Vincenzo Romano Spica, Giuliana Valerio, Matteo Vitali, Francesca Gallè

**Affiliations:** 1 Department of Movement, Human, and Health Sciences, University of Rome “Foro Italico”, 00135 Rome, Italy; federica.valeriani@uniroma4.it (F.V.); vincenzo.romanospica@uniroma4.it (V.R.S.); 2 Department of Public Health and Infectious Diseases, “Sapienza” University of Rome, 00185 Rome, Italy; carmela.protano@uniroma1.it (C.P.); daniela.marotta@uniroma1.it (D.M.); matteo.vitali@uniroma1.it (M.V.); 3 Department of Movement Sciences and Wellbeing, University of Naples “Parthenope”, 80133 Naples, Italy; giorgio.liguori@uniparthenope.it (G.L.); giuliana.valerio@uniparthenope.it (G.V.)

**Keywords:** obesity, overweight, children, adolescent, exergames, active games, weight loss

## Abstract

In the last decade, active video games (exergames) have been proposed in obesity prevention and treatment as a potential tool to increase physical activity. This review was aimed to assess the possible role of exergames in reducing weight-related outcomes among overweight/obese children and/or adolescents. The databases PubMed, Scopus, Web of Science and SPORTDiscus were interrogated to detect controlled studies involving healthy overweight/obese children and adolescents in interventions based exclusively on exergames. Out of a total of 648 articles found, 10 met the inclusion criteria and were included in the review. The included studies differ for duration, setting and type of intervention, frequency of active game sessions, and outcomes considered. Seven out of ten studies reported better outcomes in children/adolescents involved in the interventions, with significant differences between groups in four, while three studies found better outcomes in control groups. These results suggest a possible positive effect of active video games on weight-related outcomes in obese children and adolescents. However, further research is still needed to define if they can be effectively used in childhood obesity treatment and which may be the most effective approach. The potentiality of the new digital media in this field should be explored.

## 1. Introduction

In the last four decades, the prevalence of obesity among children and adolescents aged 5–19 years increased from 0.8% to 6.8% globally [1]. The World Health Organization (WHO) estimated that 124 million children and adolescents were obese in 2016 [2]. Childhood obesity is associated with early markers of cardiovascular disease, insulin resistance sleep disorders, increased risk of fractures, menstrual irregularities in adolescent girls, and negative psychological effects [2,3]. Furthermore, obese children and adolescents are more likely to become obese adults and to encounter disability and premature death than normal weight peers [2,3]. Overweight and obesity are caused by an energy imbalance which results from an increased intake of energy-dense foods and an inadequate energy expenditure through physical activity. These diet and inactivity patterns are strictly related to environmental and societal factors which facilitate the adoption of unhealthy behaviors, such as the availability of foods rich in sugars and fat or the increase of sedentary activities [4,5]. In particular, children and adolescents spend most of their daily time in a sitting position, not only during the school time but also dedicating their free time to screen-based activities, such as watching TV and playing video games [6]. This scenario was worsened by the ongoing COVID-19 pandemic, which caused many changes in dietary behaviors and physical activities of children and adolescents. Recent evidence on this issue demonstrated an increased food intake and unhealthy food choices (i.e., potatoes, meat and sugary drinks) and a significant reduction in physical activities due to the COVID-19 restriction [7,8].

Prevention and treatment of childhood obesity are mainly focused on the promotion of both community-based policies, such as taxing unhealthy foods or increasing physical exercise at school, and individual behavioral changes such as reducing excess calorie intake or screen time [3]. In recent years, exergames (or active video games) have been proposed in obesity prevention and treatment as a potential tool to increase physical activity [6,9,10]. Exergames are video games that require physical activity to interact with images on a screen through a technology based on the tracking of body movements, contrarily to passive video games that are played in a sitting position [11]. Many studies have analyzed the effects of exergames on children and adolescents’ physical activity level or energy expenditure, suggesting that they can decrease sedentary time and anthropometric measures and increase metabolic equivalents, oxygen consumption and heart rate [6,9,10,11]. However, available evidence highlights the need of further results related to specific age, gender, or weight status population groups [11]. In particular, a clear evidence about the effectiveness of exergames in determining weight loss in pediatric obesity is still lacking. Three recent reviews have systematically analyzed the effectiveness of exergames on health-related outcomes [9,10,12]. However, the review by Höchsmann et al. considered only studies performed in overweight/obese adults [12]. The reviews by Lamboglia et al. [9] and Zeng and Gao [10] were focused on youth, but the first did not consider exclusively overweight/obese children, while the second also included studies reporting other health and behavioral outcomes than weight-related ones. Furthermore, both these reviews included studies performed in the laboratory or without a control group, and the randomized controlled studies examined reported contrasting results [9,10]. Therefore, to clarify the possible role of exergames in improving weight status among overweight/obese children and adolescents, we performed a systematic review of the available literature focused on this particular item. In order to obtain more specific and valid findings, only controlled intervention studies were included in the analysis.

## 2. Materials and Methods

### 2.1. Selection Protocol and Search Strategy

The present review was conducted according to the Preferred Reporting Items for Systematic Review and Meta-Analysis (PRISMA) guidelines to identify articles on the validity of exergames as a tool for weight improvement among overweight/obese children or adolescents. The protocol was registered in PROSPERO (reference number CRD42021237839).

The review question was framed using PICOS framework and the following eligibility criteria were used: (a) Population: overweight/obese children and adolescents (age range 0–18 years); (b) Intervention: weight management interventions based exclusively on the adoption of exergames; (c) Comparison: age- and condition-matched control group; (d) Outcome: variables related to weight status (weight and/or body mass index/z-scores and circumferences reduction); Study: randomized and non-randomized controlled trials.

The databases PubMed, Scopus, Web of Science and SPORTDiscus were interrogated using the following terms: (exergam* OR “active video game” OR “active game” OR “fitness game” OR gamercise OR gamercize OR “physical game”) AND (child* OR kid OR young OR adolescent OR teen* OR youth) AND (obes* OR overweight). The search on PubMed was carried out by the title, abstract, and MeSH terms; the search on Scopus and Web of Science included topic by the title, abstract and keywords; the search on SPORTDiscus included all words.

### 2.2. Inclusion and Exclusion Criteria

The review was focused on childhood overweight/obesity. Thus, only studies involving healthy children and adolescents aged 0–18 years presenting overweight/obesity status were considered eligible. Studies including adults, underweight or normal weight subjects, individuals with further health conditions or diseases than overweight/obesity, individuals undergoing other behavioral interventions, diet or therapies for weight loss were excluded. Only articles presenting controlled trials were considered as eligible, while review, meta-analysis, semi-experimental, proceedings, qualitative and case studies, editorials, commentaries and other studies not reporting original data were excluded. Peer-reviewed literature written in English was considered, without time limitation. The search was performed until 28 February 2021.

Titles and abstracts acquired from the search were transferred to the reference site Covidence—Better systematic review management [13] for relevance assessment process. Potentially eligible studies were firstly screened by title and abstract to evaluate if they met the inclusion criteria by four authors (F.G., C.P., F.V., D.M.) independently. Then, full-texts were read independently by the same four authors (F.G., C.P., F.V., D.M.) and a decision was made about their inclusion in the review. Disagreements were achieved by consensus among the authors.

### 2.3. Data Extraction Process and Quality Assessment

A set of categories were chosen from consensus of all authors, and the following data were systematically extracted for each eligible article: bibliographic information, study design, study subject/population, type of intervention and related control. As for the results, the following weight- and body composition-related outcomes were extracted if available: weight, Body Mass Index (BMI), and/or corresponding z-scores; fat and/or muscle mass and/or corresponding percentage values; hip and waist circumference, hip and waist circumferences ratio. The quality assessment process was performed by the use of the tool for Quality Assessment of Controlled Intervention Studies designed by the National Heart, Lung, and Blood Institute’s (NHLBI) [14]. As reported previously [15,16], an overall rating of “poor,” “fair,” or “good” quality was assigned to each eligible article according to the proportion of criteria met, as follows: poor: <30% criteria met (corresponding to high risk of bias); fair: 30%–60% criteria met (some risk of bias); good: >60% criteria met (low risk of bias). Four authors (F.G., C.P., F.V., D.M.) independently assigned a score to each study, and disagreements were settled by consensus among all the authors. The Cohen’s κ value was calculated to estimate the interrater agreement for quality and risk of bias assessment.

## 3. Results

Study Selection Process and Description

**Figure 1:**
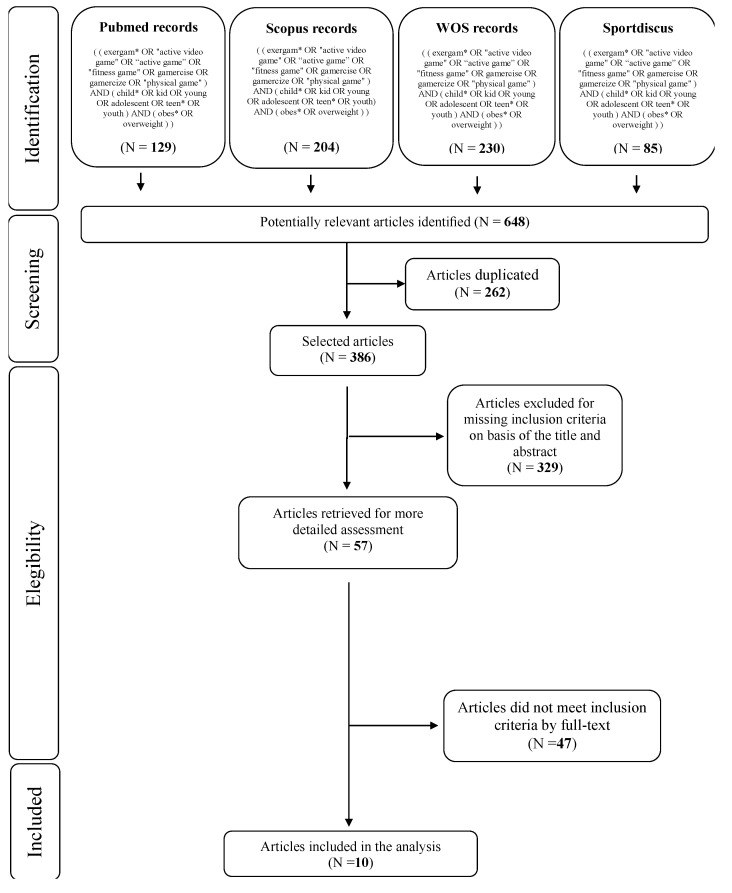
Flow-chart of search strategy.

Figure 1 shows the steps of the study selection process for the systematic review, following the PRISMA statement [17].

In total, 648 studies were identified from all searched databases and, after removing the duplicates, 386 articles were left for the following steps. Thus, of the remaining studies, 329 were deleted after analyzing the title and abstract. There were 57 articles retrieved for more detailed assessment, which have been evaluated considering the inclusion/exclusion criteria. After the evaluation of the full-text, 47 articles were excluded for the following main reasons: (i) subjects > 18 years, (ii) underweight or normal weight subjects, (iii) missing outcome between pre- and post-intervention, (iv) not a controlled trial, (v) multidisciplinary intervention. Finally, 10 articles met the inclusion criteria and were included in the analysis [18,19,20,21,22,23,24,25,26,27].

Table 1 shows the main characteristics of the selected studies.

The included articles were published between 2008 and 2020 and were performed in several countries: four trials were conducted in the USA [22,23,26,27], two in New Zealand [18,20], one in the UK [19], one in China [20], one in Canada [24], and one in the Netherlands [25]. Only the study by Liang et al. was quasi-experimental and did not randomly assign participants to the intervention and control groups [20]. All the studies included male and female subjects. The duration of the interventions ranged 6–40 weeks. Three studies were based on supervised interventions: two were performed in the school setting [19,20] and one in a clinic [27]. The quality of eight studies was evaluated as good [18,19,21,22,23,25,26,27], fair for one [20] and poor for another [24]. The Cohen’s κ value for interrater agreement was 0.94.

The main results of the selected studies in terms of outcomes related to weight status are reported in Table 2.

The study by Foley et al. reported an increase in BMI and in zBMI after 24 weeks of intervention and a reduction in body fat in both intervention and control group. Although the outcomes were better in the intervention group, no significant differences were reported [18].

In the study by Lambrick et al., participants to the 6-week intervention showed a significant increase in muscular mass and a significant decrease in waist circumference, together with non-significant increase in weight and fat mass and non-significant decrease in BMI, body fat, hip and waist:hip circumference ratio [19]. Similar, not significant trends were observed in control group, with the exception of hip circumference and waist:hip circumference ratio.

Liang et al. did not find changes in BMI in both intervention and control group after 8 weeks; a slight increase was registered for zBMI in the control group; a decrease in body fat was found in both groups, mainly but not significantly among participants to the intervention [20].

The study by Maddison et al. showed better outcomes in participants than in controls for all the considered variables but fat-free mass after 24 weeks of exergaming. Differences between the two groups were all significant, with the only exception of waist circumference change [21].

Maloney et al. reported non-significant weight loss in both intervention and control groups, with a greater decrease in control children after 12 weeks [22].

In their study, Murphy et al. observed a slight BMI decrease with a corresponding lower weight increase in participants respect to controls [23]. However, differences between times and groups were not significant.

In the 12-week pilot study by Ni Murchu et al., differences in weight (p = 0.9) and in waist circumference (p = 0.04) were found between groups [24]. However, due to the limited number of participants (*n* = 20), the authors of this pilot study argued that it may had no sufficient power to highlight differences in anthropometric outcomes.

Simons et al. reported a significant decrease of BMI-SDS in controls, with no corresponding changes in the intervention group after 40 weeks [25].

In the 24-week study by Staiano et al., significant reductions in weight and BMI z-scores, together with non-significant improvements of all the variables considered but fat mass, were registered in participants’ group respect to the control group [26].

Lastly, Wagener et al. found a minor reduction of BMI z-score in intervention and control groups, with no significant differences after 10 weeks [27].

Considering those variables that may be less influenced by age, gender and growth, an overall reduction in fat mass was reported in all the studies that measured this outcome [18,19,20,21,26], while BMI z-score decreased in three interventions [21,26,27] out of six that evaluated this variable.

Due to the variability or the unavailability of the weight-related outcomes considered in these studies, we did not perform a meta-analysis on these results.

## 4. Discussion

This review was aimed to systematically assess the current evidence regarding the effects of exergames on weight-related outcomes in childhood obesity.

Previous research has shown that active video games may be an effective method of promoting independent physical activity and limiting the time spent in a sedentary position during inactive video games [10,28]. Studies comparing exergames with other type of physical exercise found that active games increased energy expenditure, corresponding to mild, moderate, or even intensive physical activity levels, and improved physiological parameters. Exergames may support sedentary individuals to become more active, especially those experiencing difficulties or embarrassing when practicing exercise in public, as though youth with obesity [28]. However, their exclusive role in improving weight status as a consequence of this increase in energy expenditure has not been defined yet.

Our review included controlled interventions published until 2021, which were based exclusively on exergame adoption. Notwithstanding the strictness of the inclusion criteria, the selected studies were not homogeneous for duration, setting and type of intervention, nor for frequency of active game sessions and outcomes considered. Most importantly, their results were not consistent. Seven out of ten studies reported better outcomes in children/adolescents involved in the interventions [18,19,20,21,23,24,26]. Four of them detected significant differences between groups [21,23,24,26] and one showed significant improvements in the intervention group [19]. Three studies detected better outcomes in control groups [22,25,27], one of which detected significant change [25]. A reduction in the proportion of fat mass was registered in the half of the examined interventions [18,19,20,21,26], while the other five did not measure this variable. A decrease in BMI z-score was detected in three studies [19,24,25] out of six that considered this outcome.

In 2013, a systematic review was performed by Lamboglia et al. to analyze the use of exergaming against childhood obesity [9]. Exergaming was shown to increase physical activity levels, energy expenditure, maximal oxygen uptake, heart rate, and to reduce waist circumference and sedentary screen time. However, the limited number of articles available, related to the novelty of the issue, limited the validity of these findings and did not allow the authors to perform a statistical analysis on the results.

In 2015, Bochner et al. published a meta-analysis exploring the impact of active video games on weight in youth [29]. Although baseline weight condition of participants was not characterized in all the 7 randomized controlled studies selected, no difference in pre–post intervention weight change between exergaming and no-intervention groups was detected. The authors hypothesized that this was due to compensating behaviors adopted by children involved in active video games, such as increasing daily energy intake or decreasing traditional physical activity, as previously reported [30,31]. This suggests that exergaming may play its beneficial role on body weight by replacing sedentary activities such as watching television rather than substituting real exercise.

In 2016, Zeng and Gao tried to analyze the available literature on the possible effectiveness of active video games on health-related outcomes in overweight/obese individuals [10]. Of the seven randomized controlled studies and the comparison study that they analyzed for adiposity outcomes, four reported positive effects of exergaming on BMI, body composition, or body fat. The authors concluded that this type of videogame leads to a more active lifestyle by promoting physical activity and decreasing sedentary behaviors in overweight or/and obese children and youth, and can reduce body fat. Nevertheless, the Authors stated that the limited number and the quality of the available studies did not allow to highlight whether or how playing an exergame results in sustained changes in weight status and physiological responses, and the duration of occurred changes.

Unfortunately, although the majority of the selected studies seems to support the effectiveness of active video games in weight management, the results of our review do not allow to resolve these doubts so far. To date, the available literature is still insufficient and heterogeneous to express definitive conclusions.

The limitations of this review are related with these issues. First of all, the analyzed studies differ in duration and frequency of the exergaming interventions. This limited their comparison and the characterization of the effects of time and exercise volume. Exergaming, so as traditional exercise, may require a long enough period to impact body weight. Therefore, it is possible that the length and the volume of the intervention may have a role in determining its effectiveness. Nevertheless, it should be noted that children and adolescents are engaged in physical growth. It is possible that the longer the duration of the intervention, the greater might have been the influence of growth on weight-related outcomes. With regard to this, fat mass and BMI z-score can represent more reliable outcomes to evaluate changes in adiposity, but they have not been considered in all the examined interventions.

Furthermore, even the approach to exergames was different in the selected studies: some of them encouraged their use instead of inactive video games at home [18,21,22,23,24,25,26], relying on participants’ compliance, while other included supervised sessions [19,20,27]. Although no clear differences between these approaches were observed, this highlights the importance of considering the context where the intervention is performed, since several setting-related environmental, technological, and organizational factors may influence its effectiveness [32]. Furthermore, existing exergames do not offer workouts adjusted to individual fitness and are normally stationary. From this perspective, the new digital media may offer promising solutions such as smartphone-based mobile games that can be played everywhere, increasing exercise opportunities [33].

Active video games offer interesting research fields to be explored by researchers and healthcare professionals engaged in childhood obesity treatment. Due to the importance of fighting sedentarism in the management of childhood obesity, which represents a particularly critical issue during the ongoing COVID-19 pandemic, they might represent a useful tool to prevent prolonged sitting in obese youth, and to counteract possible compensatory behaviors induced by exercise programs. However, it is not yet possible to define to what extent its role may be useful in weight management, and which may be the most effective approach. The availability of further controlled studies reporting similar interventions and measuring outcomes that take into consideration the growth process is needed to achieve a valuable comparison and clear conclusions.

In the meantime, a prudent approach represents probably the best strategy in this field. As suggested by the Italian Society for Pediatric Endocrinology and Diabetology and the Italian Society of Pediatrics, the systematic use of active video games for weight loss and improvement of body composition should not be recommended but neither discouraged because they represent an additional strategy to reduce sedentary behaviors and may be useful to pursue other effects, such as an improvement in vascular response, heart rate and VO_2_max or obesity-related comorbidities, and positive psycho-behavioral and psycho-social effects [34].

## 5. Conclusions

The results of this review suggest a possible positive effect of active video games on weight-related outcomes in obese children and adolescents. However, due to the limited number and the diversity of the available studies, it is not yet possible to define if they can be effective enough to sustain weight loss and improve body composition in childhood obesity treatment.

From a public health perspective, considering the wide acceptability of exergames among youth, they may represent a useful tool to fight sedentarism and promote physical activity in obese children and adolescents. The continuous progress in new technologies opens up new horizons in this field. Therefore, further high-quality research is needed, focusing on new digital game potentiality.

## Figures and Tables

**Table 1:** Main characteristics of the selected studies.

Author, Journal, Year, Country	Study Design	Subjects	Intervention	Study Duration	Setting	Comparison	Quality Assessment
Foley et al. [18], Int J Behav Nutr Phys Act 2014, New Zealand	randomized controlled study	322 subjects aged 10–14 years) Intervention group: 160 subjects (44 F, 116 M); Control group: 162 subjects (43 F, 119 M)	Sony PlayStation Eye-Toy. Frequency: not reported.	24 weeks	home	no intervention	9/14 good
Lambrick et al. [19], J Sport Sci 2015, UK	randomized controlled study	Intervention group: 28 children (mean age 9.3 ± 0.9; 18 M, 10 F); 13 normal weight and 15 (10 M, 5 F) obese children; Control group: 27 children (mean age 9.3 ± 0.8; 14 M, 13 F): 13 normal weight and 14 (7 M, 7F) obese children	Frequency: twice-weekly 1-h exercise sessions. Children were physically active for 40-min each session, with a minimum of 48-h recovery period between sessions.	6 weeks	school	no intervention no additional exercise sessions	11/14 good
Liang et al. [20], Int J Environ Res Public Health 2020, China	quasi-experimental controlled study	Intervention group: 30 children (age 10.5 ± 0.7, 80% M); Control group: 57 children (10.4 ± 0.8, 53% M)	Xbox 360 Kinect^TM^. Frequency: two 1 h sessions/week	8 weeks	school	no intervention	8/14 fair
Maddison et al. [21], Am J Clin Nut, 2011, New Zealand	randomized controlled study	Intervention group *N* = 160—aged 11.6 ± 1.1), 72.5% boys; Control group *N* = 162—aged 11.6 ± 1.1, 73.5% boys	Hardware and gaming upgrade (Sony PlayStation 3, Kinetic, Sport, and Dance Factory). Frequency: 60 min on most days of the week.	24 weeks	home	no intervention normal video game play	12/14 good
Maloney et al. [22], Games Health J 2012, USA	randomized controlled study	Intervention group: 33 children (mean age 12.9 ± 2.36; 20 M, 13 F); Control group: 31 children (mean age 11.73 ± 2.38; 10 M, 21 F)	Sony PlayStation 2 (Dance Dance Revolution). Frequency: an average of 89 min/week	12 weeks	home	no intervention pedometer only	12/14 good
Murphy et al. [23], Int J Pediatr Obes 2009, USA	randomized controlled study	35 overweight children (7–12 years; 17 F, 18 M; BMI ≥ 85th percentile) Intervention group: 23 subjects; Control group: 12 subjects	Sony PlayStation 2 (Dance Dance Revolution). Frequency: 5 days per week (10 min/session for the 1st week, 15 for the 2nd week, 20 for the 3rd week, 25 for the 4th week and 30 for the 5th–12th week).	12 weeks	home	no intervention no additional exercise sessions	12/14 good
Ni Mhurchu et al. [24], Int J Behav Nutr Phys Act 2008, Canada	randomized controlled study	Twenty children (mean ± SD age = 12 ± 1.5 years; 40% female) randomized in intervention and control group	Active video game upgrade package for Sony PlayStation Eye-Toy, and dance mat. Frequency: encouraged substitution of inactive with active video game.	12 weeks	home	no intervention	3/14 poor
Simons et al. [25], Plos one 2014, The Netherlands	randomized controlled study	Intervention group: 134 subjects—aged 13.7 (1.3), 90% boys; Control group: 126 subjects—aged 14.1 (1,3), 92% boys	PlayStation Move (Sport Champions, Move Fitness, Start the Party and Medieval Moves, Dance Star Party and Sorcery). Frequency: as much as possible and for at least 1 h per week.	40 weeks	home	no intervention	12/14 good
Staiano et al. [26], Pediatr Obes 2018, USA	randomized controlled study	46 subjects (11.2 ± 0.8 years, 46% F) Intervention group: 23 children; Control group: 23 children	Kinect and Xbox 360 (Your Shape: Fitness Evolved 2012, Just Dance 3, Disneyland Adventures, and Kinect Sports Season 2). Frequency: 1 hr/session, 3 times/week, and weekly/beweekly videochat sessions with a fitness coach (telehealth coaching).	24 weeks	home	no intervention	14/14 good
Wagener et al. [27], Pediatr Obes 2012, USA	randomized controlled study	40 obese adolescents aged 12–18 years (66.7% female): Intervention group: 21 subjects; Control group: 20 subjects	Supervised group dance-based exergame. Frequency: 3 times a week for 40 min (including two separate 15-min exergaming segments) the first session and 75 min (including four 15-min exergaming segments) for subsequent sessions.	10 weeks	clinic	no intervention no modifications in baseline activity levels	11/14 good

**Table 2:** Outcomes related to weight status reported for participants and controls by the selected studies.

Author, Journal, Year, Country	Outcome	Intervention Group			Control Group		
		Baseline Value	Final Value	Δ	Baseline Value	Final Value	Δ
Foley et al. [18], Int J Behav Nutr Phys Act 2014, New Zealand	BMI	25.64 ± 4.08	25.85	0.11	25.75 ± 4.25	26.19	0.44
	BMI z-score	1.26 ± 1.14	1.27	0.01	1.25 ± 1.1	1.34	0.09
	BF%	32.12 ± 6.51	31.13	−0.99	32.48 ± 6.39	32.32	−0.16
Lambrick et al. [19], J Sport Sci 2015, UK	Weight (kg)	48.9 ± 11.0	49.9 ± 11.3	1	46.7 ± 11.2	49.7 ± 11.6	3
	BMI	23.7 ± 3.6	23.6 ± 3.7	−0.1	23.2 ± 3.8	22.9 ± 3.9	−0.3
	BF%	33.7 ± 7.1	32.7 ± 7.0	−1	30.4 ± 8.6	29.5 ± 8.4	−0.9
	MM (kg)	17.1 ± 3.4	18.1 ± 2.2	1 *	16.6 ± 3.0	16.7 ± 3.2	0.1
	FM (kg)	15.9 ± 5.9	16.0 ± 6.3	0.1	15.9 ± 7.6	16.0 ± 6.9	0.1
	HC (cm)	82.9 ± 9.4	81.1 ± 7.2	−1.8	81.6 ± 9.7	82.0 ± 9.0	0.4
	WC (cm)	73.2 ± 10.2	70.9 ± 8.7	−2.3 *	71.8 ± 10.2	71.6 ± 11.9	−0.2
	W:H ratio	0.88 ± 0.06	0.87 ± 0.07	−0.01	0.87 ± 0.08	0.87 ± 0.09	0
Liang et al. [20], Int J Environ Res Public Health 2020, China	BMI	18.4 ± 4.0	18.4 ± 4.0	0	18.2 ± 3.3	18.2 ± 3.3	0
	BMI z-score	0.4 ± 1.4	0.4 ± 1.3	0	0.3 ± 1.2	0.4 ± 1.2	0.1
	BF%	20.6 ± 8.5	19.6 ± 7.5	−1	19.7 ± 6.6	19.1 ± 6.7	−0.6
Maddison et al. [21], Am J Clin Nut, 2011, New Zealand	Weight (kg) ^§^	63.0 ± 13.6	63.3 ± 15.2	−0.3	63.3 ± 15.2	64.8 ± 14.4	1.5
	BMI ^§^	25.6 ± 4.1	24.8 ± 3.6	−0.8	25.8 ± 4.3	25.8 ± 4.2	0
	BMI z-score ^§^	1.3 ± 1.1	1.1 ± 1.1	−0.2	1.3 ± 1.1	1.3 ± 1.0	0
	BF% ^§^	32.1 ± 6.5	29.8 ± 7.2	−2.3	32.5 ± 6.4	31.1 ± 6.3	−1.4
	FM (kg) ^§^	20.5 ± 7.2	19.0 ± 7.1	−1.5	20.8 ± 7.6	20.3 ± 7.0	−0.5
	WC (cm)	87.3 ± 10.5	84.4 ± 10.8	−2.9	88.0 ± 10.8	88.0 ± 10.7	0
	FFM (kg) ^§^	42.2 ± 8.1	43.5 ± 7.6	1.3	42.4 ± 9.5	44.1 ± 8.9	1.7
Maloney et al. [22], Games Health J 2012, USA	Weight (lbs)	187.1 ± 67.5	185 ± 65.5	−0.2	148.8 ± 43.8	144.8 ± 61.1	−0.4
Murphy et al. [23], Int J Pediatr Obes 2009, USA	Weight (kg) ^§^	62.5 ± 15.3	63.4 ± 15.5	0.9	69.5 ± 17.0	71.9 ± 16.6	2.4
	BMI	27.9 ± 4.8	27.8 ± 5.0	−0.1	31.8 ± 5.0	32.1 ± 4.9	0.3
Ni Mhurchu et al. [24], Int J Behav Nutr Phys Act, 2008, Canada	Final weight Δ between groups (kg) BMI Final WC Δ between groups (cm) ^§^	−0.13					
		20.4 ± 3.6	-	-	19.0 ± 3.6	-	-
		−1.4					
Simons et al. [25], Plos one 2014, The Netherlands	BMI z-score	0.48 ± 1.2	0.49 ± 1.1	0.01	0.35 ± 1.1	0.28 ± 1.0	−0.7 *
Staiano et al. [26], Pediatr Obes 2018, USA	Weight z-score ^§^	2.28 ± 0.69	2.18 ± 0.74	−0.10	2.29 ± 0.65	2.33 ± 0.70	0.04
	BMI z-score ^§^	2.06 ± 0.46	2 ± 0.43	−0.06	2.10 ± 0.42	2.07 ± 0.39	−0.03
	FM (kg)	30.4 ± 11.6	31.2 ± 12.1	0.8	44.1 ± 3.4	45.8 ± 3.9	1.7
	FM%	42.0 ± 5.9	41.5 ± 6.3	−0.5	29.3 ± 7.4	29.0 ± 7.8	−0.3
Wagener et al. [27], Pediatr Obes 2012, USA	BMI z-score	3.15 ± 0.19	3.13 ± 0.18	−0.02	3.15 ± 0.20	3.12 ± 0.20	−0.03

Δ: difference between final and baseline value; BMI: Body Mass Index; FM: Fat Mass; BF: Body Fat; MM: Muscle Mass; HC: Hip Circumference; WC: Waist Circumference; * statistically significant difference between times; ^§^ statistically significant difference between groups in time.

## Data Availability

No new data were created or analyzed in this study. Data sharing is not applicable to this article.
